# MicroRNA-155 is upregulated in ascites in patients with spontaneous bacterial peritonitis

**DOI:** 10.1038/srep40556

**Published:** 2017-01-11

**Authors:** Philipp Lutz, Mohamed M´haimid, Alessandra Pohlmann, Jennifer Lehmann, Christian Jansen, Robert Schierwagen, Sabine Klein, Christian P. Strassburg, Ulrich Spengler, Jonel Trebicka

**Affiliations:** 1 Department of Internal Medicine I, University of Bonn, Sigmund-Freud-Strasse 25, 53127 Bonn, Germany; 2 German Center for Infection Research, Partner site Bonn-Cologne, Bonn, Germany; 3 Institute of Clinical Research, Faculty of Health Sciences, University of Southern Denmark, Campusvej 55, DK- 5230 Odense M, Denmark

## Abstract

MircoRNA’s (miR) have been recognised as important modulators of gene expression and potential biomarkers. However, they have been rarely investigated in bio fluids apart from blood. We investigated the association of miR-125b and miR-155 with complications of cirrhosis. Ascites was prospectively collected from patients with cirrhosis undergoing paracentesis at our department. miR’s were determined in the supernatant using qPCR and normalized by SV-40. Clinical parameters were assessed at paracentesis and during follow-up. 76 specimens from 72 patients were analysed. MiR’s were not associated to age, sex or aetiology of cirrhosis. MiR-125b levels differed between patients with low and high MELD score, and miR-125b levels showed an inverse correlation to serum creatinine (r^2^ = −0.23; p = 0.05). MiR-155 was elevated in patients with spontaneous bacterial peritonitis (SBP) (n = 10; p = 0.04). MiR-155 levels differed between patients with and without 30-day survival (p = 0.02). No association of ascites levels of investigated miR’s to size of varices, episodes of gastrointestinal bleeding or hepatorenal syndrome was observed. While miR-125b levels in ascites seem to be associated with liver and renal dysfunction, miR-155 might be implicated in local immune response in SBP.

Cirrhosis, as end-stage of any chronic liver injury, is estimated to be the fourth most common cause of death in central europe^1^. Cirrhosis is characterized by fibrosis and distortion of vascular architecture^1^ leading to increased hepatic resistance^2^, which together with splanchnic hyperperfusion^3^ causes portal hypertension. Portal hypertension is the driver of complications, such as variceal bleeding and renal dysfunction^4^. Renal dysfunction and splanchnic vasodilation contribute to ascites formation^1^. Ascites is often complicated by spontaneous bacterial peritonitis (SBP), most often caused by bacterial translocation from the intestine^5^, which might cause renal failure and is associated with high mortality.

The existence of microRNA (miR) was first reported in 1993^6^. They were described as small, non-coding RNA’s of about 21–25 nucleotides^7^. They regulate gene expression by binding to messenger RNA (mRNA)^8^ and are thereby implicated in essential biological processes, among them liver disease^8^ and inflammation^9^,^10^. In addition, due to their size and ability to circulate they are regarded as potential biomarkers for a variety of diseases including liver disease^11^,^12^,^13^,^14^. While serum levels of miR’s are accepted biomarkers for liver disease, their levels and the potential role as biomarkers in other bio fluids such as ascites are unknown in cirrhosis.

MiR-125b and miR-155 are both involved in macrophage activation^10^,^15^, which are next to lymphocytes the predominant cell population in ascites^16^. In murine peritoneal macrophages, miR-125b was upregulated after induction of peritonitis and re-stimulation with lipopolysaccharide (LPS); it was identified as a potential mediator of tolerance to LPS^17^. MiR-155 expression increased in peritoneal immune cells in a murine peritonitis model after bacterial challenge^18^ and after intraperitoneal LPS injection^19^. In addition, miR-155 has been shown to be important for antibacterial defence^20^. Therefore, we analysed the ascites levels of these two miR’s for the first time in humans as potential biomarkers for complications in cirrhosis, in particular SBP.

## Patients and Methods

### Patients

Ascites samples from patients with cirrhosis in whom a diagnostic paracentesis was performed at the Department of Internal Medicine I of the University Bonn were prospectively collected from March 2012 to September 2013. The study was approved by the local ethics committee of Bonn University Medical Centre and carried out according to the local guidelines and regulations. Prior to patient inclusion, written informed consent was obtained. Patient samples were only included if ascites was due to cirrhosis.

Diagnosis of cirrhosis was confirmed by liver biopsy, where available, or based on the presence of clinical signs of portal hypertension (oesophageal varices, splenomegaly and ascites), appropriate findings in ultrasound and standard laboratory. Age, sex, aetiology of cirrhosis, Child-Pugh stage, model for end-stage liver disease (MELD) score, standard laboratory parameters and complications of cirrhosis (spontaneous bacterial peritonitis, hepatorenal syndrome, hepatic encephalopathy, variceal bleeding, hepatocellular carcinoma) were recorded. Patients were followed-up for a maximum of 365 days. Patients who received a liver transplant were considered dead at the day of transplant. Diagnosis of hepatorenal syndrome and grading of esophageal varices was done according to current international guidelines^21^,^22^.

Ascites specimen of patients with cirrhosis were classified according to international guidelines^21^ in ascites without infection, SBP or control after SBP. Bacterascites was defined by the detection of bacteria in the presence of a normal ascites PMN count.

## Methods

Standard laboratory parameters (serum albumin, bilirubin, creatinine, C-reactive protein, INR, sodium, total blood count, ascites total protein, ascites leukocyte and ascites PMN count) were measured in the central laboratory of the university hospital of Bonn with routine procedures. Ten mL of the ascitic fluid were delivered at the bedside into aerobic and anaerobic blood culture bottles (BD BACTEC, Becton Dickinson Heidelberg, Germany) and incubated for a maximum of 5 days in a Bactec FX blood culture system (Becton Dickinson) for microbial studies. Ascites samples were centrifuged at 300 g for 10 minutes. Then the supernatant was stored at −20 °C until MicroRNA-extraction and analysis.

### Determination of miR

RNA was isolated from ascites samples using the Qiazol reagent following the instructions of the supplier (Qiagen, Hilden, Germany) as previously described^12^,^13^. SV40-miR (Qiagen) was added to the samples (2 pmol/200 μl) prior to the RNA isolation procedure for later normalization of the miR levels, while RNA quantity was determined by A_260_-measurement using the ND-1000 NanoDrop spectrophotometer (NanoDrop, Wilmington, DE, USA) and quality was assessed by microcapillary electrophoresis (2100 BioAnalyser, Agilent Technologies, Waldbronn, Germany).

MiR was analyzed by a two-step real-time PCR using the miScript-Reverse Transcription Kit and the miRNA-SYBR Green PCR Kit (Qiagen, Hilden, Germany). MiR-125b, miR-155 and SV-40 primers used for cDNA synthesis and real-time PCR were selected and purchased from the GeneGlobe Search Center (Qiagen, Hilden, Germany). All steps were performed in triplicate and in agreement with the supplier’s guidelines. For normalization of the miR levels, spike-in SV40-miRNA (Qiagen, Hilden, Germany) was used. MiR levels are expressed as 2^−ΔCT^ with ΔCT being cycle threshold (CT) of the respective miR minus CT of SV-40.

### Statistical Analysis

Data are reported as median and range, if not stated otherwise. Statistical analyses were performed with IBM SPSS Statistics software version 23 (IBM, New York, USA). For the analysis of quantitative data, Mann-U or Wilcoxon signed-rank test were used, as appropriate. Fisher’s exact test was applied to qualitative data. For correlation of quantitative data, Spearman’s rank correlation coefficient was calculated. Survival was analysed with Kaplan-Meier plots and log-rank test. Patients who received a liver transplant were considered as dead at the time of transplant. Contribution of several parameters on ascites miR-155 levels was assessed by a forward multiple linear regression model. P < 0.05 was considered significant.

## Results

### Study cohort

We included 76 ascites specimens of 72 patients with cirrhosis and ascites. The four samples acquired during follow-up from the same patients showed difference in infection status (3 times paired SBP/non-SBP and 1x paired bacterascites/normal ascites). The majority of patients were male (72%), median age was 62 years. Most patients had cirrhosis due to alcohol abuse (67%) and presented in an advanced stage of liver disease (53% in Child-Pugh stage C, median MELD score 17). Levels of miR-125b were in general higher than levels of miR-155. Further details of the patient cohort are reported in Table 1.

### Levels of miR-125b in ascites are inversely associated to a high MELD score and to serum creatinine

**Figure 1:**
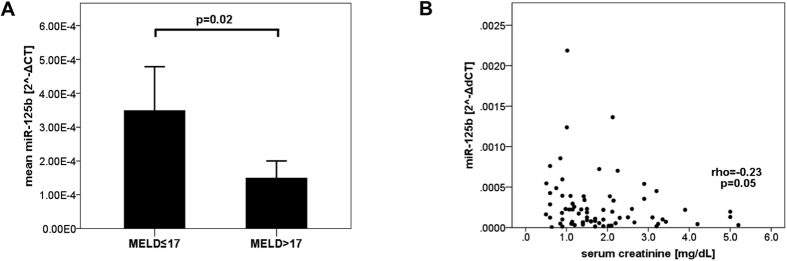
(**A**) Ascites microR-125b levels differ between patients stratified for the MELD score. Means of ascites microR-125b levels with error bars reflecting 2x standard error of the mean. MELD: model of end-stage liver disease, miR: microRNA. Statistical analysis with Mann-Whitney-U test. (**B**) Correlation between ascites miR-125b and serum creatinine miR: microRNA; Statistical analysis with spearman’s rank correlation.

We correlated the respective miR levels to patients’ age, gender, aetiology and severity of liver disease, as well as ascites protein levels (Table 2). We noted a statistical trend towards a low inverse correlation between ascites miR-125b levels and MELD score levels. Therefore, we stratified our patients according to a MELD score up to or above the median in our cohort. We observed a significant difference concerning ascites miR-125b levels between the two groups (p = 0.02, Fig. 1A). However, no difference in miR-125b level between Child-Pugh stage B and C was noted (0.17 vs 0.16 2^−ΔCT^ × 10^−3^; p = 0.93). Upon closer analysis of the relation between MELD score and miR-125b, we found a week borderline correlation between miR-125b and serum creatinine (Fig. 1B, Table 2), but not to serum bilirubin or INR levels. MiR-155 did not correlate to these parameters.

Restriction of analysis to specimens without ascites infection led to comparable results regarding gender, age, ascites total protein, MELD score up to/below 17 (p = 0.04), aetiology of cirrhosis and Child-Pugh-stage.

### Ascites miR-155 is elevated in SBP

**Figure 2:**
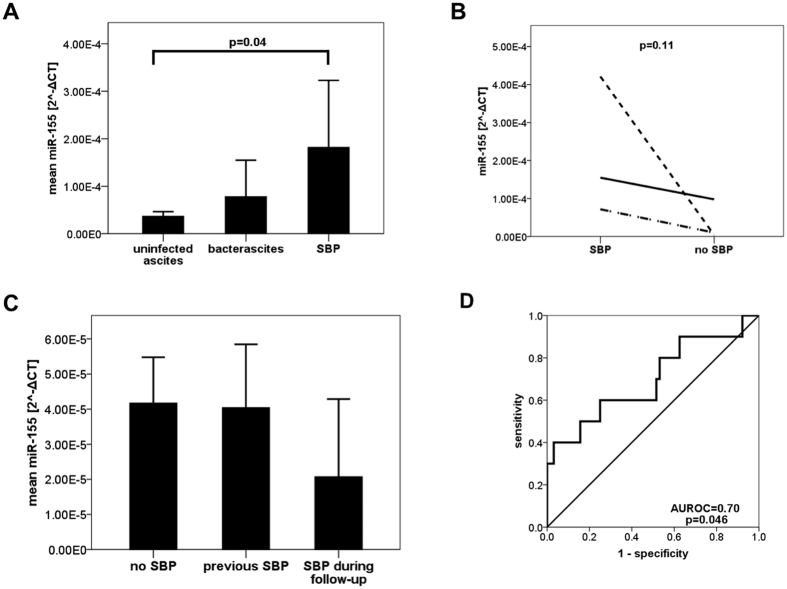
(**A**) Ascites miR-155 was elevated in patients with spontaneous bacterial peritonitis. Means of ascites microRNA-155 levels with error bars reflecting 2x standard error of the mean for uninfected ascites (n = 62), bacterascites (n = 4) and SBP (n = 10) specimens. SBP: spontaneous bacterial peritonitis; Statistical analysis with Mann-Whitney-U test. (**B**) Ascites miR-155 increased during spontaneous bacterial peritonitis intraindividually. Ascites microRNA-155 at time of SBP and without SBP. miR: microRNA; SBP: spontaneous bacterial peritonitis; statistical analysis with Wilcoxon signed-rank test. (**C**) Ascites miR-155 was comparable in patients regardless of history/future occurrence of spontaneous bacterial peritonitis. Ascites microRNA-155 levels in patients stratified for previous SBP (n = 21), no SBP (n = 39) or SBP during follow-up (n = 6). miR: microRNA; SBP: spontaneous bacterial peritonitis. Statistical analysis with Mann-Whitney-U test; p = n.s. (**D**) Receiver operator characteristic curve for miR-155 as diagnostic test for spontaneous bacterial peritonitis. miR: microRNA; AUROC: area under the receiver operator characteristic curve.

Next, we compared patients with SBP to patients without ascites infection (Table 3). In addition to higher ascites PMN counts, patients with SBP were found to have higher serum C-reactive protein levels and higher white blood cell counts compared to patients without SBP. Ascites levels of miR-125b did not differ. However, miR-155 was significantly elevated in the ascites of SBP patients (Fig. 2A). In bacterascites samples, miR-155 ranged between uninfected ascites and SBP, but failed statistical significance to either of these groups due to the small sample size of bacterascites. In every of the available three paired ascites samples between SBP and non-SBP, miR-155 decreased in non-SBP compared to SBP (Fig. 2B). In the one patient, in whom a paired sample between bacterascites and uninfected ascites was available, levels of miR-155 were identical between the two specimens (both 0.08 2^−ΔCT^ × 10^−3^). We further analysed if miR-155 levels differed in non-SBP specimens for patients with previous SBP (n = 21), no (n = 39) or future SBP (n = 6), but levels were comparable (Fig. 2C; p = 0.50). To evaluate miR-155 as potential diagnostic test for SBP, we created a receiver operator characteristic (ROC) curve. The area under the ROC curve (AUROC) was 0.70 (p = 0.046, Fig. 2D). Sensitivity was 90% when using 0.016 2^−ΔCT^ × 10^−3^ as cut-off, resulting in a low specificity of 37%.

Upon closer analysis of miR-155 levels on features and outcome of SBP patients, there was no difference between miR-155 levels between patients with culture positive (n = 4; median miR-155 0.223 2^−ΔCT^ × 10^−3^) versus culture negative SBP (n = 6; median miR-155 0.063 2^−ΔCT^ × 10^−3^; p = 0.91), between patients with rapid treatment response to SBP versus none/delayed response (n = 5 each; median miR-155 0.071 2^−ΔCT^ × 10^−3^ versus 0.055 2^−ΔCT^ × 10^−3^; p = 1.0) and between SBP patients with (n = 8) versus without (n = 2) concomitant acute kidney failure (median miR-155 0.091 2^−ΔCT^ × 10^−3^ versus 0.063 2^−ΔCT^ × 10^−3^). However, when we stratified SBP patients according to 30-day survival, ascites miR-155 levels were lower in non-survivors (n = 4) compared to survivors (n = 6, median 0.019 2^−ΔCT^ × 10^−3^ versus 0.228 2^−ΔCT^ × 10^−3^; p = 0.02).

### Ascites miR’s were not associated to hepatorenal syndrome or variceal bleeding

Next, we investigated if levels of ascites miRNA’s were associated to a history of variceal bleeding, presence of medium to large varices or hepatorenal syndrome. No statistically significant association was found (Table 4).

### Ascites miR’s and presence of hepatocellular carcinoma

**Figure 3:**
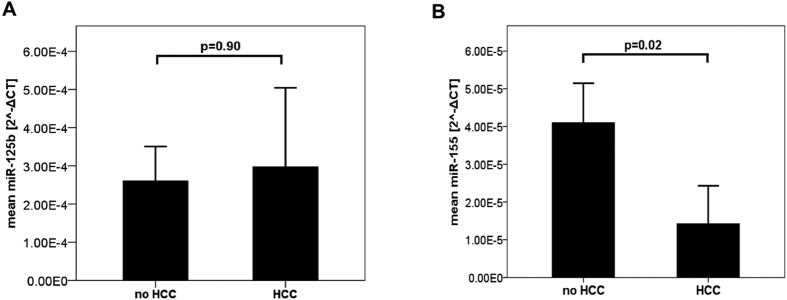
(**A**) Ascites microRNA-125b was comparable between patients with and without hepatocellular carcinoma. Means of ascites microRNA-125b levels with error bars reflecting 2x standard error of the mean for patients with (n = 14) and without (n = 58) hepatocellular carcinoma. HCC: hepatocellular carcinoma; Statistical analysis with Mann-Whitney-U test. (**B**) Ascites microRNA-155 was downregulated in hepatocellular carcinoma patients without ascites infection. Means of ascites microRNA-155 levels with error bars reflecting 2x standard error of the mean for ascites specimens without infection in patients with (n = 11) and without (n = 51) hepatocellular carcinoma. HCC: hepatocellular carcinoma; Statistical analysis with Mann-Whitney-U test.

When we analysed the association between ascites miRNA levels and presence of hepatocellular carcinoma (HCC), we found no association for miR-125b (Fig. 3A), but a statistical trend for miR-155. When we excluded patients with ascites infection, we detected significantly lower ascites miR-155 levels in patients with HCC compared to patients without HCC (Fig. 3B). In multivariate analysis, considering SBP and HCC as potential contributors to ascites miR-155 levels, only SBP was found to contribute significantly to miR-155 levels (standardized Beta: 0.494; p < 0.001).

### Survival

No significant difference in 180 day and one year survival was detected when patients were stratified according to the median of the respective miR.

## Discussion

In this pilot study, we evaluated associations of ascites miR levels to common complications of chronic liver disease. By using ascites as bio fluid, we chose a compartment located directly next to the diseased organ, which might reflect disease better than peripheral blood, but which is easily accessible in patients with cirrhosis. We analysed miR-125b and miR-155, which have been involved in macrophages activation^15^ and regulation of innate immunity responses^9^. To our knowledge, we provide the first data on ascites microRNA levels in chronic liver disease.

MiR-125b levels were different in patients with high and low MELD as indicator of liver disease severity. However, we did not detect any difference applying Child-Pugh stages as another method for assessing severity of liver disease. Since most of our patients ranged quite similar on the Child-Pugh stages, the MELD score might have been more discriminative. However, miR-125b levels correlated lowly, but significantly to serum creatinine. A negative correlation between miR-125b and kidney function has already been reported before^23^. In addition, miR-125b negatively regulates expression of the protective angiotensin-converting enzyme 2 in renal tubular epithelial cells, thereby mediating kidney damage to high glucose levels^24^. Because creatinine is one of the three parameters used to calculate the MELD score^25^, it is likely that the difference in miR levels between high and low MELD scores did not reflect an association to liver disease severity, but rather the difference in kidney function. As expected, serum creatinine levels were significantly different between high and low MELD scores in our cohort (median serum creatinine 2.5 versus 1.2 mg/dL; p < 0.001). Low blood miR-125b levels have been linked to decreased miR-125b expression in smooth vascular cells in kidney disease^23^. To what extent vascular cells account for miR-125b blood levels is not known. Although miR-125b is expressed at significantly higher amounts in macrophages compared to other immune cells and mediates activation of macrophages^15^, the contribution of macrophages to systemic or peritoneal miR-125b levels might be low compared to vascular cells. Additionally, miR-125b expression in macrophages might be the results of complex opposing effects in patients with cirrhosis, who usually have elevated LPS levels^26^, because first exposure to LPS down-regulates miR-125b in macrophages^17^,^27^, but re-stimulation to LPS up-regulates miR-125b expression, linking miR-125b to LPS tolerance^17^. SBP is a trigger for the development of renal failure^28^,^29^, as well as circulatory and inflammatory changes are preceding the development of organ failure in context of acute-on-chronic liver failure^30^, although the exact mechanism is not fully understood. MiR-125b might be one of the mediators, which might be linked to kidney dysfunction in the situation of bacterial translocation. Although the present study is far from proving this link, it might strongly suggest it and initiate further research.

Ascites miR-155 was significantly upregulated during SBP in our patients. Similar to our observation in humans, establishing peritonitis by intraperitoneal injection of Klebsiella pneumoniae resulted in local upregulation of miR-155 in mice^31^. Upregulation was more pronounced in a similar peritonitis mouse model after pre-treatment with LPS^18^, which might mimic the state of enhanced bacterial translocation of a cirrhotic patient before SBP develops^26^. MiR- 155 increased after peritonitis induction and was again associated with immune dysregulation in another mouse model assessing the effect of hypothermia on immune dysregulation^19^. MiR-155 has been involved in various mechanism of immune response, comprising maturation of B lymphocytes, responsiveness of B lymphocytes to B-cell receptor ligation^32^, promotion of a Th1 phenotype^33^, enhancing antibacterial defense mechanisms of CD8 T cells^34^, proliferation of myeloid cells and inflammatory cytokine secretion^9^. In MiR-155 knock-out mice, immunity against enteric infections such as Salmonella and Citrobacter^35^ is impaired. Interestingly, miR-155 is not only induced by activation of extracellular receptors for bacterial products, such as toll-like receptor 4, but also by intracellular receptors such as nucleotide-binding oligomerization domain-containing protein 2 (NOD2)^36^. Polymorphisms in the *NOD2* gene have been linked to susceptibility to SBP and to mortality in patients with cirrhosis and ascites^37^. Therefore, miR-155 might be an immunological mechanism which is initiated early in the response to ascites infection and therefore represent a novel marker of SBP. Interestingly, low miR-155 levels during SBP were associated with decreased short time survival. Relatively low miR-155 levels during SBP might reflect inability of the host to mount an adequate immune response to bacterial infection and thus explain the unfavourable outcome. Still, these findings must be validated and investigated in further studies.

MicroRNA’s are considered to be potential drug targets by anti-microRNA oligonucleotides^38^. As a proof of concept, anti-miR-122 has been applied safely in patients with chronic hepatitis C and shown excellent treatment success^39^. Inhibition of miR-155 was feasible in a mouse modell^40^. Further investigation of the role of miR-155 in peritoneal immune response and immune dysregulation in cirrhotic patients will be eminent to evaluate its role as a potential drug target that would even allow local application into the ascites in patients with SBP.

Dysregulation of microRNA’s is a common finding in malignant tumours^41^ and has been reported for hepatocellular carcinoma^42^. The particular role of microRNA’s, however, seems to be complex and depending on the tumor entity. For example, miR-125b has been shown to be upregulated in tissue of gastric cancer and to enhance spread of gastric cancer metastases^43^, but to inhibit formation of metastases in hepatocellular carcinoma^44^. We did not aim to assess a potential relation between ascites microRNA and malignancy in our study. Still, we found an association between miR-155 and presence of hepatocellular carcinoma, which, however, was lost upon multivariate analysis. Ascites is not a suitable bio fluid to screen for hepatocellular carcinoma, because treatment options are limited once patients have developed ascites due to advanced liver disease^45^.

We present a single centre pilot study on ascites miRNA. Further studies including larger patient numbers are needed to confirm our findings.

In conclusion, we provide first data on ascites microRNA levels in patients with advanced liver disease, indicating that microRNA’s might be implicated in the pathogenesis of bacterial ascites infections. If our results can be confirmed in larger patient series, miR-155 might be an interesting target to modify immune response during bacterial infections in patients with cirrhosis.

## Additional Information

**How to cite this article**: Lutz, P. *et al*. MicroRNA-155 is upregulated in ascites in patients with spontaneous bacterial peritonitis. *Sci. Rep.* **7**, 40556; doi: 10.1038/srep40556 (2017).

**Publisher's note:** Springer Nature remains neutral with regard to jurisdictional claims in published maps and institutional affiliations.

## Figures and Tables

**Table 1:** Characteristics of the patients’ cohort.

	Patient cohort
Patient number [n]	72
Age [years ]	62 (56; 69)
Male sex [n]	49 (72)
Aetiology of cirrhosis:	
-Alcohol	48 (67)
-Viral hepatitis	9 (13)
-Alcohol + viral Hepatitis	3 (4)
-Other	12 (16)
Child-Pugh-Score A/B/C [n]	1 (1)/33 (46)/38 (53)
MELD score	17 (13; 24)
Medium-large esophageal varices [n]	20 (28)
History of variceal bleeding [n]	18 (27)
History of hepatorenal syndrome [n]	58 (85)
Previous SBP [n]	24 (33)
Hepatocellular carcinoma [n]	14 (19)
Bilirubin [mg/dl]	2.0 (1; 3.7)
Creatinine [mg/dl]	1.6 (1; 2.5)
International normalized ratio	1.3 (1.1; 1.5)
Platelets [G/L]	150 (112; 228)
Sodium [mmol/L]	136 (133; 140)
Ascites total protein [g/L]	11 (7; 24)
Specimens - without infection/bacterascites/SBP [n]	62 (82)/4 (5)/10 (13)
miR-125b [2^−ΔCT^ 10^−3^]	0.2 (0.06; 0.4)
miR-155 [2^−ΔCT^ 10^−3^]	0.03 (0.01; 0.06)

CT: cycle threshold; MELD: modell for end-stage liver disease; miR: microRNA; SBP: spontaneous bacterial peritonitis.

Data are given as median (lower; upper interquartile) or absolute number (percent).

**Table 2:** Association of ascites miR levels to age, gender, severity and aetiology of liver disease and ascites protein levels.

		miR-125b [rho]	MiR-155 [rho]
Age		0.03	0.04
Ascites total Protein		0.04	0.13
Serum bilirubin		−0.10	−0.13
Serum creatinine		−0.23*	−0.03
INR		0.001	−0.009
MELD		−0.20^+^	−0.004
		**miR-125b [2^−ΔCT^ × 10^−3^]**	**MiR-155 [2^−ΔCT^ × 10^−3^]**
Gender	male/female	0.12/0.18	0.03/0.03
Aetiology	alcoholic/viral	0.1/0.2	0.03/0.04

^+^p < 0.1; ^*^p = 0.05; CT: cycle threshold; INR: International normalized ratio; MELD: model for end-stage liver disease; miR: microRNA.

Data are given as spearman’s correlation coefficient or as median. Statistical analysis with spearman’s rank correlation or Wilcoxon-Mann-Whitney-U test.

**Table 3:** Clinical markers of inflammation and ascites miR levels in patients with and without spontaneous bacterial peritonitis.

	Uninfected ascites	SBP	p
Number of specimens	62	10	
Culture positive	0	4 (40)	
Detected microorganisms	0	2 × Escherichia coli	
		1 × Enterobacter cloacae	
		1 × Enterococcus faecium	
C-reactive protein [mg/dL]	17 (11; 32)	104 (60; 174)	<0.001
White blood cell count [G/L]	7.6 (6.2; 10.5)	9.5 (8.5; 13.6)	0.02
Ascites PMN count [/μL]	24 (9; 54)	1237 (552; 3947)	<0.001
MiR-125b [2^−ΔCT^ × 10^3^]	0.16 (0.06; 0.37)	0.11 (0.06; 0.24)	0.59
MiR-155 [2^−ΔCT^ × 10^3^]	0.03 (0.01; 0.05)	0.06 (0.02; 0.4)	0.04

CT: cycle threshold; MELD: model for end-stage liver disease; miR: microRNA; SBP: spontaneous bacterial peritonitis.

Data are given as median (lower; upper interquartile) absolute number (percent). Statistical analysis with Mann-Whitney-U test.

**Table 4:** Association of ascites miR levels to variceal bleeding, size of esophageal varices and hepatorenal syndrome.

		**miR-125b** [2^−ΔCT^ × 10^−3^]	**miR-155** [2^−ΔCT^ × 10^−3^]
History of variceal bleeding	yes/no	0.1/0.19	0.03/0.04
Esophageal varices	small/medium-large	0.23/0.14	0.04/0.02
History of hepatorenal syndrome	yes/no	0.17/0.15	0.03/0.03

Data are given as median. Statistical analysis with Mann-Whitney-U test.
